# Distribution of Non-Tuberculous Mycobacterium Species in Pulmonary and Extrapulmonary Infections in South India–A Retrospective Analysis

**DOI:** 10.3390/microorganisms14061319

**Published:** 2026-06-12

**Authors:** Priya Rajendran, Radha Gopalaswamy, Gowsalya Saminathan, Bershila Prey, Hannah Stanley, Adhin Bhaskar, Sudha Solayappan, Dinesh Viswanathan, Radhakrishnan Ramalingam, Asha Frederick, Sivakumar Shanmugam

**Affiliations:** 1 Department of Bacteriology, ICMR–National Institute for Research in Tuberculosis, Chennai 600031, India; priya.raj@icmr.gov.in (P.R.); bershilaprey@gmail.com (B.P.); r.radhakrish@gmail.com (R.R.); 2 Department of Statistics, ICMR–National Institute for Research in Tuberculosis, Chennai 600031, India; 3 Directorate of Medical and Rural Health Services, Chennai 600006, India

**Keywords:** non-tuberculous mycobacteria, extrapulmonary NTM, line probe assay, *M. abscessus*, *M. kansasii*

## Abstract

Differential diagnosis of tuberculosis (TB) and non-tuberculous mycobacteria (NTM) is extremely challenging, especially in a high TB burden setting like India. A definitive diagnosis of NTM, along with additional speciation, is warranted to improve NTM management. Beyond the diagnosis of NTM and its speciation, clinical correlation is vital for differentiating NTM colonisation or contamination from disease. In this cross-sectional, retrospective analysis, both pulmonary and extrapulmonary samples from 1121 presumptive NTM patients from Tuberculosis Units across the country and from other private hospitals were included. Composite diagnosis were performed using X-rays, nucleic acid amplification tests, smear microscopy, and mycobacterial growth indicator tube cultures, with speciation of NTM isolates confirmed by line probe assay. Of the 1121 presumptive NTM patients, 66.0% were smear-negative, 44.7% had X-ray changes, and 98.0% were *M. tuberculosis*-negative according to the nucleic acid amplification test. Cultures identified 310 patients as NTM-positive, including 22 extrapulmonary cases. Speciation was performed for 135 NTM-positive isolates, where *M. abscessus* was identified as the predominant species in 30.4%, followed by *M. kansasii* in 25.1%. Our study demonstrated that although the composite diagnosis of NTM holds promise for identifying pulmonary and extrapulmonary NTM, culture (mean confirmation rate of 30–40% over 5 years) remains the gold standard, with NTM speciation by line probe assay completing the diagnosis.

## 1. Introduction

Non-tuberculous mycobacteria (NTM) constitute a large, heterogeneous group of acid-fast bacilli distinct from the *Mycobacterium tuberculosis* complex (MTBC) and *M. leprae*. More than 200 species and subspecies have been identified, and these organisms are widely distributed in natural and engineered environments, including soil, water distribution systems, and hospital plumbing [1]. Although regarded as contaminants or opportunistic pathogens earlier, NTM are increasingly recognised as clinically significant causes of pulmonary and extrapulmonary disease in recent times [2]. In contrast to tuberculosis (TB), which is predominantly transmitted from person to person, NTM infections are often environmentally acquired [3]. This fundamental difference complicates surveillance, as classical TB transmission-control strategies do not directly affect NTM disease. Instead, its burden reflects the interplay of environmental exposure, host susceptibility, and diagnostic awareness [4]. Over the past two decades, population-based analyses have documented a steady increase in NTM pulmonary disease (NTM-PD) worldwide [1].

India, with its high TB burden, faces a dual challenge: the overlap of TB and NTM presentations and the need to strengthen laboratory networks for both. This constraint emphasises the importance of accurate and rapid diagnosis of NTM for effective patient management. Conventional phenotypic methods, based on growth rate, pigmentation, and biochemical tests, can aid in species identification; however, these methods are often unreliable and time-consuming [5]. In contrast, molecular and proteomic tools have transformed diagnostic capacity in recent years. Line probe assays targeting species-specific genes, MALDI-TOF mass spectrometry, and sequencing of conserved regions such as *hsp65*, *rpoB*, or 16S rRNA provide rapid species-level identification. Whole-genome sequencing (WGS), although currently limited to reference centres, provides the highest resolution, enabling species and subspecies identification as well as insights into resistance determinants and epidemiological links [6].

NTM species differ significantly in their pathogenic potential, clinical relevance, and drug-susceptibility profiles, underscoring the need for species-level identification. While some isolates, such as *M. gordonae*, are typically contaminants or colonisers, others, such as the *M. avium* complex (MAC) and *M. abscessus*, have clear therapeutic implications. Without proper species identification, clinicians risk both overtreating colonisers and underdiagnosing true infections. Epidemiological studies consistently demonstrate the influence of host demographics on NTM disease, and the interaction between species variation and host demographics is clinically important [7]. While older age groups with COPD and prior TB are more likely to present with *M. kansasii* or fibro cavitary MAC disease, a middle-aged group without a smoking history may develop nodular-bronchiectatic MAC disease [8,9]. Recognising these demographic patterns helps interpret positive cultures, distinguish colonisation from infection, and tailor therapy.

Standardised reporting of NTM species and patient demographics through expanded diagnostic algorithms could yield valuable epidemiological insights. This is particularly relevant in India, where high TB prevalence and prior TB lung damage may predispose large populations to NTM infection. With this context, the present study aims to provide epidemiological insights into microbiologically confirmed pulmonary NTM under the National TB Elimination Program (NTEP) framework. By retrospectively analysing species diversity and patient demographics (age, sex), this work seeks to bridge gaps in local epidemiology, facilitate accurate clinical interpretation of NTM isolates, and inform both therapeutic decision-making and public health surveillance.

## 2. Materials and Methods

### 2.1. Study Design and Setting

Sputum and extrapulmonary samples taken between January 2019 and December 2024 from 1121 presumptive NTM patients referred by Tuberculosis Units (TUs) across the country and other private hospitals for the confirmation of NTM and speciation were included in this five-year cross-sectional retrospective analysis. They were transported to the ICMR-National Institute for Research in Tuberculosis, a National Reference Laboratory (NRL) under the NTEP.

Inclusion criteria include (1). Symptomatic patients; (2). Chest X-ray positive, indicative of mycobacteria; (3). Smear-positive or negative/Cartridge-based nucleic acid amplification testing (CBNAAT) MTB negative, and (4). Smear-positive/Immunochromatographic test (ICT negative among culture-positive patients whose CBNAAT status is unknown or MTB negative). Exclusion criteria: patients with CBNAAT-positive for MTB.

This study was approved by the Ethics Committee of the ICMR-National Institute for Research in Tuberculosis (NIRT-IEC ID 2022-017), Chennai, India. As this study involved a retrospective data analysis, a waiver of written informed consent was obtained from the NIRT-IEC.

### 2.2. Study Method

Sputum was processed using the standard N-acetyl-L-cysteine (2.9%) and sodium hydroxide (4%) (NALC-NaOH) decontamination method. The specimen was treated with an equal volume of NaLC-NaOH for 15 min, neutralised with PBS, and centrifuged at 3000× *g* for 20 min at 4 °C to collect the processed deposit [10]. Extrapulmonary samples were processed according to the guidelines issued by the NTEP and the Central TB Division (CTD), New Delhi [11]. The processed samples were subjected to smear microscopy with fluorescent staining to identify acid-fast bacilli (AFB), and then inoculated into the MGIT 960 (Becton Dickinson and Company, Gurgaon, Haryana, India) liquid culture system [10]. For cultures positive on the MGIT 960 system, contamination was checked on Brain Heart Infusion agar (BHIA) (Sigma-Aldrich, Bengaluru, India) by spotting the same and incubating at 37 °C for 24–48 h. Additionally, the presence or absence of MTBC was identified using MPT-64 (Abbott India, Mumbai, India) and AFB bacilli were visualised using Ziehl-Neelsen (ZN) staining on an LED microscope (Zeiss Primostar New Delhi, New Delhi, India).

### 2.3. NTM Speciation

For smear-positive cultures that were MPT-64-negative, they were declared NTM, and speciation was performed using line probe assay (LPA). The DNA was extracted from the cultures using the Genolyse kit (Hains Lifesciences, Nehren, Germany), and the samples were then subjected to LPA using the NTM CM kit (Hains Lifesciences, Nehren, Germany). NTM speciation was interpreted according to the manufacturer’s instructions.

### 2.4. Data Collection

Patient data were collected from laboratory records and the electronic web-enabled Ni-Kshay (Ni = End, Kshay = TB) portal established by the CTD, Ministry of Health and Family Welfare, India. The data included age, gender, NAAT results, X-ray radiographic results, and sample types. Comorbidities (HIV, Diabetes), social habits (current tobacco and alcohol use), and weight and height were also obtained. Data abstraction was conducted until July 2024 to update patient status.

### 2.5. Operational Definitions

NAAT Mtb Detected–MTB Detected using GeneXpert MTB/RIF (Cepheid, Sunnyvale, CA, USA) or Truenat MTB-RIF (Molbio Diagnostics, Verna, Goa, India)
NAAT Mtb Not Detected–MTB Not Detected using GeneXpert MTB/RIF or Truenat MTB-RIF
X-ray positive–X-ray changes indicative of mycobacterial disease
Smear-positive–Smears of sputum or Extrapulmonary sample deposit positive (Scanty; 1+; 2+ and 3+ grades) by fluorescence microscopy
Smear-Negative–Smears of sputum or an extrapulmonary sample deposit negative by fluorescence microscopy
Culture Negative–Cultures not showing any growth after 42 days of incubation were considered negative
Culture NTM-positive–Cultures positive with smear-positive; No growth on BHIA and MPT-64 negative
Culture MTB positive–Cultures positive with smear-positive; No growth on BHIA and MPT-64 positive
Culture contaminated—Culture-positive flagged by MGIT, but there is growth on BHIA, with both smear and MPT64 being negative.

### 2.6. Statistical Analysis

Data were analysed using SPSS version 25 (Statistical Package for the Social Sciences Inc., Chicago, IL, USA). Variables are expressed as frequencies, percentages, medians, and interquartile ranges. A chi-square test was performed to assess the association between the variables.

## 3. Results

**Figure 1:**
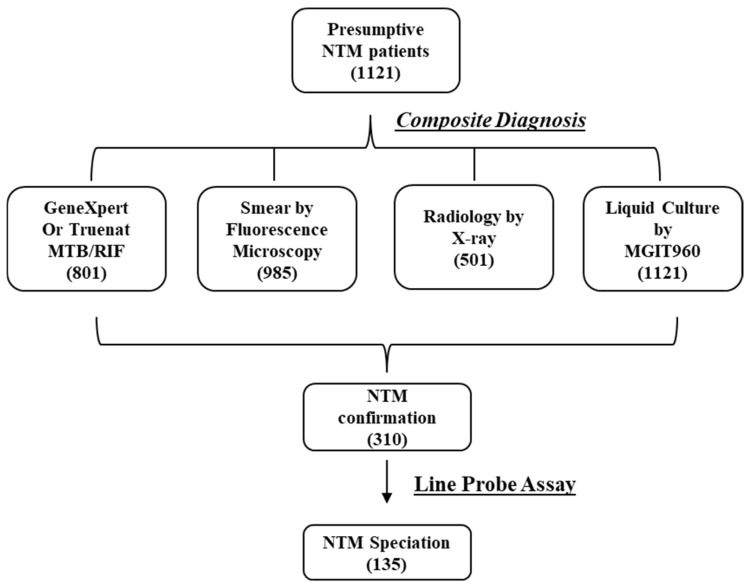
The flowchart illustrates the total number of presumptive NTM patients and their composite diagnosis based on the tests offered (indicated by numbers). A line probe assay was performed for NTM speciation.

A total of 1121 presumptive NTM patients were referred to ICMR–NIRT for confirmation and speciation of NTM from January 2019 to December 2024. Of these 1121 patients, 642 provided more than two samples, as per IDSA guidelines, and of the remaining 479 samples, 73 were extrapulmonary. The patients underwent a composite diagnosis using NAAT, chest X-ray, smear microscopy, MGIT culture, and LPA, wherever possible (Figure 1). Culture and LPA were used at the NRL to confirm NTM diagnosis, followed by speciation. The tests offered for the number of patients are presented in Figure 1.

### 3.1. Baseline Characteristics

The available data on the patients’ baseline characteristics, consolidated from the records, are presented in Table 1. Overall, among the 1118 patients with available gender data, 61.3% were male, and 38.7% were female. The median age of the patients was 51 years (IQR, 51–60 years), with the majority of the study population falling within the 41–59 years age range (41.7%). Among presumptive NTM patients, 92.5% had pulmonary infections and 7.5% had extrapulmonary infections. A previous history of TB was reported for 10.9% of the total. When the comorbidities were explored, 4.8% were HIV-reactive and 20.4% were diabetic. 16.8% of patients had a current history of tobacco use, with 14.0% of them smokers. Alcohol abuse was reported in 19.5% of patients.

### 3.2. Laboratory Diagnosis for Presumptive NTM Patients

Symptom screening, chest X-rays, and NAAT were offered onsite as initial tests at the field level. NAAT testing ruled out MTB in 785 of 801 patients and 501 patients had abnormal chest X-rays, prompting further laboratory investigations. Fluorescent microscopy using auramine staining was performed on 985 pulmonary and extrapulmonary samples, with 34.0% (335/985 patient samples) of them being AFB-positive (Table 2). Of the 1121 samples cultured, 59.9% (671/1121) were negative. A total of 450 patients were culture-positive and underwent further identification by smear microscopy using ZN stain. An ICT card was used to rule out MTBC, and brain heart infusion agar (BHIA) spots were used to detect contamination. Among culture-positive patients after contamination was ruled out, 81.3% (310/381) were identified as NTM, including 22 patients with extrapulmonary TB, while 18.7% were confirmed as MTBC.

The sociodemographic and clinical characteristics of the patients, along with culture results, are presented in Table 3. Except for Chest X-ray (*p* = 0.02), no significant association was observed between NTM and these characteristics.

### 3.3. Species Identification and Distribution

Of 310 patients identified as having NTM by culture, 135 could be speciated with the NTM CM/AS kit (Table 4). Of these 135 patients, 14 had a mixed NTM infection, including MTBC. In comparison, 121 patients had an NTM infection caused by a single infectious agent. The predominant NTM species identified were *M. abscessus* in 30.4% (41/135) of patients and *M. kansasii* in 25.1% (34/135). The distribution of NTM species among patients is shown in Table 3, where uncommon species, such as *M. peregrinum* and *M. genavense*, were identified in mixed infections. Among 14 extrapulmonary NTM isolates, five had *M. abscessus*, four had *M. intracellulare*, and three had a mixed infection with two NTM species, each of which provided an accurate diagnosis in paucibacillary samples. In addition, when the distribution of MTB and NTM was compared over the years, NTM consistently exceeded MTB, with the highest incidence in 2021.

## 4. Discussion

There has been a steady increase in the incidence of NTM infections over the past decade, attributed to several factors, including increased awareness among healthcare workers and advancements in microbiological diagnostic methods. Unlike MTB, NTM does not rely on a single regimen; treatment varies by species [12]. The patients with pulmonary symptoms, X-ray abnormalities, and NAAT-negative results are considered clinically diagnosed TB, especially in TB-endemic regions, and are subjected to MTB treatment [13]. However, the differential diagnosis of these cases to rule out NTM in such scenarios has been encouraged recently. As part of this initiative, our lab has been collecting pulmonary and extrapulmonary samples from patients suspected of having NTM infections across various TB units and private hospitals to support patient management. We conducted a retrospective analysis of data obtained from these patients, focusing on demographic characteristics, laboratory test results, and species distribution among the NTM-positive isolates.

With composite diagnostic tests offered at the onsite and reference labs, the percentage of NTM isolation among 1121 patients was 27.6% (310). When the data from initial diagnostic tests were compared among 1121 patients, 335 out of 985 tested were smear-positive, and 785 of 801 tested were NAAT-negative out. A study in China demonstrated that smear-positive NAAT-negative results can accurately predict the existence of NTM in clinical specimens [14]. However, in our study, we could not rely on this correlation because only 89 of 335 smear-positive patients were NTM- and NAAT-negative. Moreover, there were more NTMs (168) in smear and NAAT-negative samples. Out of these 168 samples, nearly 50% (86) had chest X-ray abnormalities, and there was also a significant association with overall NTMs isolated (*p* = 0.02). This suggests that radiological diagnosis can be utilised as a screening tool for presumptive NTM patients with NAAT-negative results. With the replacement of smears with upfront NAAT testing as the initial diagnostic test for TB, a composite diagnosis of NTM involving NAAT-negative results, chest X-ray findings, and NTM culture from at least two samples or from a single sterile site of infection as per ATS/IDSA guidelines will help estimate the true burden of NTM [12]. There was no significant association between baseline characteristics and other risk factors and NTM isolation. This could be because as part of the program, factors predominantly at risk for TB were collected and did not include a data set indicating risk of NTM (structural lung disease, genetic factors, and surgery). However, the higher NTM isolation rate in males than in females and in the 18 to 60-year-old age group, warrants special focus, because studies have shown that age and gender are the major demographic risk factors for NTM infection [15,16]. Similarly, although the associations between other risk factors, like smoking, alcohol, and NTM, are not significant, studies have demonstrated that they may play a major role in treatment outcome [17,18]. Another major risk factor that needs attention is that 19 (8.0%) out of 310 patients detected with NTM had a previous history of TB. Studies have documented that the proportion of NTM infections with a previous history of TB is high in India [19,20].

Previous studies indicate that 20.0–30.0% of NTM infections originate from extrapulmonary sites [21]. In our study, we documented extrapulmonary NTM involvement in 7% of patients (22/310). However, a higher percentage (19.4%) of NTM extrapulmonary infections was documented in another south Indian study. This could be because the study included NTM identification among presumptive TB patients, as well as cultures referred for speciation from pulmonary and extrapulmonary specimens [16]. Similarly, a multicentric study spanning the northern, western, and southern regions of India also documented 17.2% extrapulmonary involvement among NTM patients [22]. The lower NTM level in our study may be mainly due to the predominance of NAAT-negative pulmonary samples, with fewer extrapulmonary samples referred to the study. Given that extrapulmonary infections due to NTM have increased in recent years, continuous monitoring of NTM and improved referral of presumptive NTM patients of both pulmonary and extrapulmonary sites is a pressing need of the hour.

Identification and speciation of NTM by LPA using Hains CM/AS are considered superior due to their high accuracy, rapid turnaround time, and ease of use in programmatic settings compared with more sophisticated methods such as tNGS, MALDI-TOF, and WGS [6]. Previous studies have shown the sensitivity of the GenoType Mycobacterium CM/AS assay to be 98.1–99.4%, with the specificity of 98.3–100% when biochemical tests, including PNB or SD strip tests, were used as the standard [23,24]. Hence, we employed this speciation method for the NTM isolates obtained from culture in our study.

The species distribution of pulmonary NTM diseases in Asia is primarily attributed to the *M. avium* complex (*M. avium* and *M. intracellulare*) and *M. abscessus* [3]. A recent study from north India also documented *M. avium* complex (25.6%) as the predominant species, followed by *M. abscessus* (24.7%) [22]. Similarly, a study from South India documented the predominance of *M. intracellulare*, followed by *M. abscessus* (17.7%) and *M. kansasii* (12.7%) [16]. In contrast, *M. abscessus* (30.4%) was the predominant species in our study, followed by *M. kansasii* (25.1%) and *M. intracellulare* (14.0%). This could be attributed to the fact that, in addition to program samples, we have been receiving samples from tertiary care centres and private hospitals. Studies have shown that *M. abscessus* is more commonly associated with hospital infections due to its resistance to many glutaraldehyde-based hospital-grade disinfectants and various antibiotics [25,26]. This could be related to a study from a tertiary care centre in north India, in which a high prevalence of *M. abscessus* (31.3%) was documented, followed by *M. fortuitum* (22.0%) [27].

Among the mixed infections, one patient with MTBC and two fast-growing species, *M. fortuitum* and *M. peregrinum* (no previous history of TB, MTB not detected by CBNAAT, Chest X-ray abnormal) was identified, underscoring the complexity of NTM diagnosis compared with MTBC. Previous studies involving MTBC and NTM have indicated a potential risk of overlooking mixed infections, as only MTBC was considered, reiterating the need for diagnostic evaluation, epidemiological investigation, and clinical consideration [28,29,30]. We speciated only 135 of 310 NTM cultures due to inconsistent resources and poor recovery of stored cultures. There could be bias in how availability and recovery were used to select cultures for speciation, which contributed to a major limitation in this retrospective analysis.

The ATS/ERS/ESCMID/IDSA clinical practice guideline outlines diagnostic and treatment considerations for NTM infections. Diagnosis is usually preceded by clinical and radiological evidence of pulmonary disease, exclusion of alternative diagnoses, and microbiological confirmation of NTM in more than 2 sputum samples or in a single bronchial wash sample [12,13,31]. Unlike pulmonary disease, extrapulmonary NTM infection presents with clinical features that depend on the site of infection and typically include fever, pain, inflammation, nodules, lesions, ulcers, and skin/soft tissue infections [21,32]. The treatment of an NTM infection depends on the site of disease, the clinical assessment of pathogenicity, the benefits of therapy, and the patient’s willingness. Watchful waiting is recommended based on the clinician’s decision, though specific drugs are prescribed at specific doses and duration depending on the species identification. While clarithromycin is recommended for *M. avium* complex (along with amikacin or streptomycin) and *M. abscessus*, rifampicin, isoniazid, and ethambutol are recommended for *M. kansasii*, *M. malmoense*, and *M. xenopi*, and azithromycin or amikacin, fluoroquinolones, and doxycycline are recommended for *M. fortuitum* and *M. chelonae* [12,21]. Mixed NTM infections, though rare, are clinically challenging owing to the similarity in pulmonary symptoms, making differentiating between colonisation and actual disease-causing NTM during diagnosis difficult. Treatment typically includes drugs against one or more NTM involved, requiring a personalised regimen that may include clarithromycin, moxifloxacin, imipenem, cefoxitin, amikacin, azithromycin, ethambutol, isoniazid, and rifabutin, with the aim of alleviating symptoms and clinical improvement, alongside culture conversion [33,34,35].

Given the significant role of NTM species in pulmonary manifestations and extrapulmonary infections, appropriate and rapid speciation to the subspecies level is essential for patient management. Moreover, clinical correlation would be encouraged before treatment to rule out colonisation rather than actual NTM disease in every case. There is a paucity of country-wide distribution data for NTM, warranting a multicentric study with unbiased coverage across geographic regions, inclusion-exclusion criteria, proportionate pulmonary and extrapulmonary samples, consistent testing and confirmation of NTM and speciation with uniform interpretation, clinical correlation to confirm NTM disease, and a better understanding of the prevalence and distribution of NTM species.

## 5. Limitations

1. We could not retrieve cultures from all smear-positive and NAAT-negative samples, and with that loss, the true prevalence rate of NTM could not be estimated.
2. Being a retrospective study, we could not do a systematic analysis of the data since for some of the patients, one or another set of data was missing.
3. The sample distribution was not even for all the years.
4. The findings are from a passive case flow from districts to the reference centre and not a community study.
5. With a lack of additional clinical correlation following the identification or detection of NTM, we have defined NTM-positive patients as patients detected or identified with NTM to indicate a lack of differentiation of colonisation or contamination from a disease, to designate all or a subset as NTM patients.
6. The NTM speciation was restricted to 135 out of 310 patients detected with NTM, indicating a selection bias where certain species will be missed out while studying distribution.

## 6. Conclusions

Our study demonstrated that a composite diagnosis of NTM holds promise for identifying both pulmonary and extrapulmonary samples, facilitating the differential diagnosis of MTB and NTM. Stringent, systematic data collection on NTM through the NTEP would not only improve clinical care but also guide national antimicrobial stewardship across NTM species. Ultimately, strengthening the evidence base for NTM in India is essential, as the global incidence of these infections continues to rise and their clinical and public health importance becomes increasingly apparent.

NTM diagnosis and treatment are very challenging, especially in programmatic settings. The following actionable recommendations can be implemented under the NTEP for patient screening, sample collection, diagnosis, recovery, clinical management, and treatment regimen planning, including prognosis and outcomes [12,18,31].

1. Screening of symptomatic patients, especially those with predisposing lung diseases like chronic obstructive pulmonary disease, bronchiectasis, and cystic fibrosis.
2. Symptomatic patients should be subjected to radiology and microbiology (smear and nucleic acid amplification tests) for the confirmation of NTM.
3. Care should be taken when collecting pulmonary samples, as sputum should be collected after rinsing the mouth with clean water to avoid contamination. For extrapulmonary samples, the affected area should be rinsed with sterile solution and the sample collected using autoclaved surgical instruments and stored in sterile saline for testing.
4. Specimen storage and transportation should be conducted at optimal temperatures and conditions to avoid contamination and enable optimal recovery of bacteria.
5. IDSA guidelines recommend positive cultures from at least two sputum samples, one bronchial wash, or one lung biopsy sample for pulmonary disease.
6. After confirmation by culture, NTM speciation should preferably be performed using molecular techniques such as line probe assays or next-generation sequencing.
7. NTM treatment for rapid and slow growers typically includes rifampicin, isoniazid, ethambutol, clarithromycin, amikacin, streptomycin, imipenem, moxifloxacin, and cefoxitin, in various multidrug combinations, with or without surgical intervention, as needed.
8. Drug susceptibility testing for common NTM drugs is essential to guide the treatment regimen.
9. Clinical correlation should be done with the history of the patient, site of infection, clinical symptoms, and NTM species identified to confirm NTM disease.
10. Treatment initiation should be guided by symptom severity, feasibility, and the patient’s willingness, and typically includes a watchful-wait phase before the decision to start treatment.
11. Treatment prognosis should be monitored by regular follow-up aiming at symptom alleviation, smear and culture conversion, and the lack of adverse effects. Long-term follow-up is required beyond completion to assess any relapse.

## Figures and Tables

**Table 1:** Baseline characteristics of presumptive NTM patients screened under NTEP from January 2019 to December 2024.

Characteristics	Description	N *	n	%
**Age (years)**	≤18	1089	33	3.1
	19–40		263	24.1
	41–59		455	41.7
	≥60		338	30.9
**Gender**	Male	1118	686	61.3
	Female		432	38.7
**Sample Type**	Pulmonary	972	899	92.5
	Extrapulmonary		73	7.5
**Previous history of TB**	No	949	845	89.1
	Yes		104	10.9
**HIV status**	Non-Reactive	916	872	95.2
	Reactive		44	4.8
**Diabetes**	Non-diabetic	901	717	79.6
	Diabetic		184	20.4
**Current tobacco use**	No	856	712	83.2
	Yes		144	16.8
**Smoking**	No	856	736	86.0
	Yes		120	14.0
**Alcohol intake**	No	852	686	80.5
	Yes		166	19.5

* Number of patients for whom the data is available. “N” denotes the total number of patients with valid results for the characteristic, with the number of patients under every classification indicated as numbers (n) and percentage (%).

**Table 2:** Laboratory Diagnosis of presumptive NTM patients.

Laboratory Testing	Patient Characteristics	N	n	% (95% CI)
**Smear**	Positive	985	335	34.0
				(31.05, 36.97)
	Negative		650	66.0
				(63.03, 68.95)
**Low Complexity NAAT**	MTB Not Detected	801	785	98.0
				(97.03, 98.97)
	MTB Detected		16	2.0
				(1.03, 2.97)
**Chest X-ray**	Positives	1121	501	44.7
				(41.78, 47.6)
	NA		620	55.3
				(52.4, 58.22)
**Culture**	NTM	1121	310	27.6
				(25.04, 30.27)
	MTB		71	6.3
				(4.91, 7.76)
	Negative		671	59.9
				(56.99, 62.73)
	Contaminated		69	6.2
				(4.75, 7.56)

“N” denotes the total number of patients with valid results for the characteristic, with the number of patients under every classification indicated as numbers (n) and percentages (%). NA–Not available.

**Table 3:** Comparison of sociodemographic and clinical characteristics with culture results (n = 802).

Variable		Culture			*p*-Value	Bonferroni-Adjusted *p*-Value
		NTM	Neg/Cont	MTB		
**Gender**	Male	141 (65.58)	366 (64.66)	29 (56.86)	0.494	1.00
	Female/TG	74 (34.42)	200 (35.34)	22 (43.14)		
**Age (years)**	<18	5 (2.33)	15 (2.65)	2 (3.92)	0.805	1.00
	18 to <60	151 (70.23)	374 (66.08)	33 (64.71)		
	60 and above	59 (27.44)	177 (31.27)	16 (31.37)		
**Sample type**	Pulmonary	194 (90.23)	525 (92.76)	49 (96.08)	0.289	1.00
	Extra pulmonary	21 (9.77)	41 (7.24)	2 (3.92)		
**Previous history of TB**	No	196 (91.16)	506 (89.4)	42 (82.35)	0.184	1.00
	Yes	19 (8.84)	60 (10.6)	9 (17.65)		
**HIV status**	No	203 (94.42)	540 (95.41)	50 (98.04)	0.537	1.00
	Yes	12 (5.58)	26 (4.59)	1 (1.96)		
**Diabetes**	No	165 (76.74)	459 (81.1)	40 (78.43)	0.388	1.00
	Yes	50 (23.26)	107 (18.9)	11 (21.57)		
**Tobacco**	No	184 (85.58)	470 (83.04)	39 (76.47)	0.281	1.00
	Yes	31 (14.42)	96 (16.96)	12 (23.53)		
**Smoking**	No	188 (87.44)	488 (86.22)	41 (80.39)	0.423	1.00
	Yes	27 (12.56)	78 (13.78)	10 (19.61)		
**Alcohol**	No	175 (81.4)	458 (80.92)	37 (72.55)	0.328	1.00
	Yes	40 (18.6)	108 (19.08)	14 (27.45)		
**X-ray abnormality**	Yes	90 (41.86)	299 (52.83)	24 (47.06)	0.022	0.22
	NA	125 (58.14)	267 (47.17)	27 (52.94)		

The total number of patients with valid results for the characteristic is provided along with the percentage within parentheses. NA–Not available.

**Table 4:** NTM speciation by NTM CM/AS Line probe assay and their distribution.

Species	Pulmonary	Extrapulmonary
**Single NTM Infection**		
***M. abscessus***	33	4
***M. kansasii***	28	1
***M. intracellulare***	19	4
***M. fortuitum***	12	0
***M. chelonae***	5	0
***M. scrofulaceum***	5	0
***M. simiae***	4	0
***M. avium***	4	0
***M. szulgai***	1	1
**Total**	**111**	**10**
**Mixed NTM Infection**		
***M. fortuitum*** **and *M. peregrinum***	3	1
***M. fortuitum*** **and *M. abscessus***	2	1
***M. avium*** **and *M. kansasii***	1	1
***M. chelonae*** **and *M. genavense***	1	0
***M. kansasii*** **and *M. intracellulare***	1	0
***M. kansasii*** **and *M. chelonae***	1	0
***M. kansasii*** **and *M. abscessus***	1	0
**MTB complex, *M. fortuitum* & *M. peregrinum***	1	0
**Total**	**11**	**3**

## Data Availability

The original contributions presented in this study are included in the article. Further inquiries can be directed to the corresponding author.

## Abbreviations

TB	Tuberculosis
NTM	Non-tuberculous mycobacteria
MTBC	Mycobacterium tuberculosis complex
TU	Tuberculosis units
NAAT	Nucleic acid amplification test
MAC	*M. avium* complex
NTEP	National TB Elimination Program
NRL	National Reference Laboratory
CBNAAT	Cartridge-based nucleic acid amplification testing
ICT	Immunochromatographic test
NaLC	N-acetyl-L-cysteine
NaOH	Sodium hydroxide
CTD	Central TB Division
AFB	Acid-fast bacilli
MGIT	Mycobacterial growth indicator tube
BHIA	Brain Heart Infusion agar
ZN	Ziehl-Neelsen
LPA	Line probe assay
NA	Not available
